# Challenges in the Accurate Surveillance of Booster Seat and Bicycle Helmet Usage by Children: Lessons from the Field

**DOI:** 10.3390/ijerph13070658

**Published:** 2016-07-07

**Authors:** Curt Pankratz, Lynne Warda, Caroline Piotrowski

**Affiliations:** 1 Department of Sociology, University of Winnipeg, Winnipeg, MB R3B 2E9, Canada; 2 Injury Prevention and Child Public Health Program, Winnipeg Regional Health Authority, Winnipeg, MB R3A 1S1, Canada; lwarda@exchange.hsc.mb.ca; 3 Department of Pediatrics and Child Health, University of Manitoba, Winnipeg, MB R3A 1S1, Canada; 4 Department of Community Health Sciences, University of Manitoba, Winnipeg, MB R3T 2N2, Canada; caroline.piotrowski@umanitoba.ca

**Keywords:** child safety, traffic injuries, surveillance, data collection, booster seats, bicycle helmets

## Abstract

Motor vehicle collisions and bicycle collisions and falls are a leading cause of death by preventable injury for children. In order to design, implement and evaluate campaigns and programs aimed at improving child safety, accurate surveillance is needed. This paper examined the challenges that confront efforts to collect surveillance data relevant to child traffic safety, including observation, interview, and focus group methods. Strategies to address key challenges in order to improve the efficiency and accuracy of surveillance methods were recommended. The potential for new technology to enhance existing surveillance methods was also explored.

## 1. Introduction

Motor vehicle collisions are a leading cause of death by preventable injury for children. For this reason, creating effective awareness and prevention programs to increase children’s traffic safety, and understanding the impact of relevant legislation concerning the use of safety equipment such as booster seats and bicycle helmets are a high priority. However, there are considerable challenges associated with accurate surveillance of appropriate usage of these devices. The purpose of this paper was to examine key challenges that confront efforts to collect data regarding the awareness, perception, and use of booster seats and bicycle helmets, and to recommend strategies for effectively overcoming these challenges. Although our work has focused on the Canadian context, the practical nature of these challenges exist in any country.

## 2. The Importance of Booster Seats and Bicycle Helmets

Child safety in motor vehicles and while riding bicycles is a significant problem [1]. While the use of child restraints significantly reduces the risk of injury in vehicle collisions, children are often inappropriately or incorrectly restrained [2,3,4]. The majority of children aged 4–8 prematurely use lap and shoulder seat belt systems [5], which may lead to abdominal, spinal cord, and/or head injuries during collisions [6]. The American Academy of Pediatrics recently recommended that children use a booster seat until they fit into a lap and shoulder belt properly; that is typically when children are 4 feet 9 inches in height and between eight and 12 years of age [7]. The need for a booster seat is largely dependent on the seat structure within vehicles. Geometrical analysis has suggested that most vehicles are unlikely to produce a good lap belt fit for 75% of children aged six to 12, and that children under the age of 12 are very likely to experience poor seatbelt fit after transitioning out of a booster seat [8]. Nevertheless, most existing legislation does not mandate booster seat use beyond eight years of age (for examples, see [9,10,11]).

It has been well established that booster seat use reduces injuries through compensating for children’s small body size [3]. While it is clear that booster seats provide protection from injury and death, rates of use are estimated to be low among children four to eight years of age across Canada, the United States and Australia [12,13,14,15], all of which have recently introduced legislation mandating their use. For example, in Canada, 24.6% of four to eight-year-olds were observed to be restrained in booster seats in provinces with legislation, and 16.6% in provinces without [12]. Usage rates also vary by surveillance methodology, which may have the unintended effect of confusing parents concerning the actual degree of risk if booster seats are not used. Similar patterns are evident for children’s use of bicycle helmets.

Bicycle collisions and falls are a significant cause of traumatic brain injury in children [16]. Studies of patients presenting to hospital emergency departments found that head injuries are more common among children falling from bicycles than from other wheeled activities such as skateboarding [17]. Among children aged 5–14, who have the highest rate of bicycle-related injuries, 29% never wore a helmet while riding [18]. Bicycle-to-bicycle collisions have also been found to be prevalent, especially in urban environments [19]. Although these injuries tend to be more moderate, severe injuries can occur to the head or other extremities [19,20]. Recent research examining the risk of fatal head injuries found that not wearing a helmet while riding a bicycle increased the risk of sustaining a head injury, as well as the risk of death from such an injury [21].

Like booster seats, bicycle helmets have been found to reduce the risk of serious injury for children. For example, helmet use reduced the risk of serious head injury in bicycle collisions with vehicles by up to 74%, with more severe injuries showing the greatest reduction [22]. Recent work has also found that bicycle helmets were effective in greatly reducing the impact on the skull from momentary impact [16]. Despite these findings, bicycle helmet use is relatively low even when legislation is in place. For example, recent research has found that children’s helmet use increased from 43% to 53% two years after the implementation of mandatory use legislation using observation and survey methodology [23]. Other work using police-reported road crash and hospital admission data for injuries sustained in bicycle collisions with motor vehicles confirmed that approximately half of children under 19 years of age were not wearing a helmet [22]. Age has consistently been related to bicycle helmet use, with younger children more likely to wear them [18], both in Western countries and globally [24], and with children becoming less likely to wear helmets as they age [25]; however, as was noted with booster seats, usage rates do vary by surveillance methodology.

## 3. Surveillance

In order to effectively assess the impact of injury prevention campaigns, legislation and other safety interventions, tracking the use of bicycle helmets and booster seats must be as accurate, consistent, and comprehensive as possible. Although surveillance can be done in numerous ways, we will focus on the three most common forms of surveillance used in booster seat and bicycle helmet research: field observation, interview (in person or by telephone) and focus group discussions. Observation can be done passively (for example, watching from a public roadside), or it may include stopping vehicles, although the latter is much more time- and labor-intensive. Each of these methods allows for the collection of different kinds of information regarding the factors associated with the use of booster seats and bike helmets. At the same time, there are also serious challenges associated with each method.

### 3.1. Booster Seat Observations

Observation is one of the most common surveillance methods for monitoring the use of booster seats. In Canada, booster seat research typically uses a version of Transport Canada’s Roadside Observation protocol for child restraint surveillance, which has been employed and validated in several studies [14]. This method involves choosing an observation site along a public roadway or intersection and looking into vehicles as they are sitting in traffic, typically at a red light. This method has good face validity, is well-established and non-intrusive, in that informed consent is not required from individual participants.

Since 2010, we have conducted booster seat observations in Winnipeg as well as several sites across Manitoba using this method. These observations were conducted at 29 sites across Winnipeg, stratified by two sites within 12 neighbourhood clusters, and five additional sites near busy shopping malls on weekends. Observations were recorded by trained research assistants and, as much as possible, the same observers conducted observations each year. It was required that observed vehicles must contain at least one child estimated to be between five and eight years of age, and vehicles were excluded if the age of the child was uncertain. For each vehicle, the driver’s sex and seatbelt use were also recorded, as well as an estimate of the children’s age, their seating position and restraint status. Vehicles were used as the unit of analysis, identified as either a “user” or “non-user” because there were very few instances of vehicles with more than one child in the correct age range where only one child was using a booster seat while others were not. Therefore, the booster seat usage rates reported here were based on the number of vehicles in which children were using booster seats, rather than the number of children. Analysing the booster seat data by vehicle also aims to address the issue of lack of independence of the observations within the vehicle. In other words, if a particular parent/caregiver chooses to use booster seats, they will, if at all possible, use them for all of their children. Counting each child would inflate use rates when vehicles contain more than one child. In our observations over the years, we have recorded very few cases wherein only some of the children aged five to eight in the same vehicle were in booster seats. Booster seat use was also observed in a number of rural locations using three categories: urban, rural (within 75 km of Winnipeg) and rural-remote (more than 75 km from Winnipeg). Based on our experience with this methodology over the past several years, we have identified a number of serious challenges.

The first key challenge was that an observer must be able to accurately estimate a child’s age through a vehicle window. This challenge was compounded by the frequency of darkly tinted rear windows in minivans, which are commonly used for transporting children. In addition, the only part of a child sitting in a vehicle that is visible to an observer is from the shoulders up, excluding the use of height as an indicator of age, although researchers can be trained to better estimate age by viewing pictures of children of various ages while sitting in vehicles. This training is important and requires careful attention before sending observers into the field.

In addition to estimating the age of a child, field observers must also be able to determine whether or not a child is sitting in a booster seat. This is made more difficult by the fact that there are different kinds of booster seats, with some having backs and others being backless. Backless booster seats tend to be less costly and more common, but also more difficult to observe from outside a vehicle. Because of this challenge, identification of a booster seat was typically based on how high up a child was sitting. This challenge was amplified by the variation between different vehicle types, which can make a seated child appear higher or lower in their seat depending on window and seat height. There are some common structural factors that can be applied here, however. Booster seats are designed to elevate children to the sitting-height anticipated by vehicle layout (most notably the location of the shoulder belt and window). If a booster seat is being used correctly, a child should be seated in such a way that the shoulder belt is correctly positioned across the shoulder rather than the neck. In addition, vehicles are designed so that the average passenger can easily look out the window. If a booster seat was being used, a child therefore should be able to see out the window without reaching and looking upward. This means that accurate identification of booster seat usage can be based on the location of the shoulder belt and the extent to which a child’s face comes above the bottom of the window. These structural aspects facilitate accurate identification of booster seats usage. An additional criterion that can be used to address this challenge includes comparing the width of a child’s shoulders to their apparent elevation in their seat. This applies the fact that a child’s shoulder width is related to her/his height, and is an applicable criterion if used in conjunction with the other strategies noted above.

We found that all of the above considerations should ultimately be approached holistically when training observers. A good training approach was to have observers review a series of photos of children in vehicles and record the data for each one. Their estimates were then compared for accuracy. This allowed for ambiguous scenarios to be staged in order to test the correct identification of borderline cases. In the field, observers worked in pairs, with both recording the same vehicle and then later checking inter-rater reliability.

Another less commonly used training strategy is to compare roadside observation with other methods. For example, one pair of observers could record roadside booster seat observations near a parking lot entrance, and then compare their observations with those of other observers stationed in the parking lot who observe occupants exiting the same vehicles, or who wait until occupants have exited vehicles and look inside the vehicle for the presence or absence of booster seats. Alternatively, observers could also approach occupants as they exit their vehicle and ask them to confirm the ages of children present and the presence or absence of booster seats. Although more time- and labour-intensive, such comparison methods can help improve and validate observer accuracy and reliability. However, relying solely on parking lot surveys or other data collection methods where vehicles are stopped and asked to participate in a study or survey introduces a significant volunteer bias and overestimates booster seat rates [2]. Direct observations that include all eligible vehicles such as roadside inspections by law enforcement eliminate this bias but introduce potential ethical issues regarding voluntary participation by research participants.

**Figure 1:**
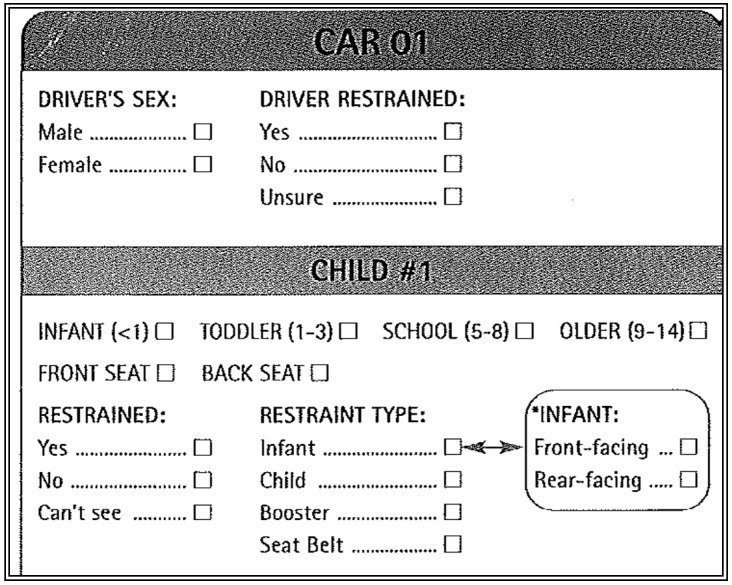
Transport Canada roadside observation for vehicles with child passengers.

In addition to using more intensive training methods to reduce observer bias, observation recording methods can also reduce bias. For example, we found that although the Transport Canada roadside observation data collection form is logically organized, it could be improved to reduce potential bias. Figure 1 shows the relevant part of the data collection form.

The form was designed to record any type of restraint use for children under the age of 15. The difficulty is that, as noted above, while it is possible to estimate if a child is using a booster seat or not using a child’s seated height in a vehicle, it is not possible to estimate if they are wearing a lap belt. The result is that if a lap belt cannot be seen, that child will be recorded as “unobservable”, even if it is obvious that the child is not using a booster seat. It is possible for this problem to bias reporting in either direction, since it is possible to know that a child is in a booster seat while still not seeing a lap belt. However, it is far less common for a child to be using a booster seat without a seatbelt.

Because of this shortcoming, the structure of the form encourages the systematic loss of cases where children are not in a booster seat. Such a bias could lead to over-estimates of booster seat use prevalence. To demonstrate how this bias might affect surveillance results, in our recent data collection we asked observers to check two boxes: “unobservable” for whether or not a child was restrained, followed by “seatbelt” for the restraint type. Upon data entry, we have taken the combination of those two selections (in other words, cases where those two boxes are checked) to mean that a child is definitely not in a booster seat, but that we are unsure of whether or not a lap belt was used. This provided important information not only concerning whether the child was wearing a lap belt, but also whether or not the child was improperly restrained due to the lack of a booster seat. It also allowed for all of the observed negative cases to be included; we have compared our observational findings with and without such cases. The results in Table 1 show booster seat use usage data collected annually from 2010–2015 with and without these hybrid cases included in the analysis.

As predicted, the removal of cases where a child was clearly not in a booster seat, but where a lap belt was not seen led to an over-estimate of booster seat use. Both data sets show a steady increase in booster seat use over time, but the disparity between the data sets increased over time as well. By 2015, the observed booster seat usage rate was 44.4% using the revised form, but was 63.0% using the original form. This large discrepancy could have serious implications for the evaluation of legislation or education campaigns.

The timing of roadside observations could be another potential source of bias. For example, booster seat observations must be done during hours when children aged four to eight years are not in school. At the same time, the school year is the time when families are most likely to be following their normal routines, which may affect the vehicles they use, where they are driving, and whether they use safety devices. Therefore, most observations are typically conducted after school and on weekends. In our research, we have used a weeknight shift of 3:30–7:30 p.m. for observing five- to eight-year-olds; however, weekend observations, as well as observations on school holidays including summer vacation, are equally important and should be included as much as possible.

### 3.2. Booster Seat Self-Report

Self-report methods are also an important source of information that provides more detailed demographic data, as well as participant perceptions and rationales that complement observational data [26]. In 2010, we conducted random digit computer-assisted telephone interviews (CATI) with Winnipeg parents (*n* = 128) of children aged four to nine to assess booster seat use patterns. Parents were contacted only at home telephone locations. Completion of the survey was voluntary and confidential. The sample consisted of English-speaking adults who were parents of at least one child in the booster seat age range. Parents were asked general demographic information, patterns of booster seat use, and about factors potentially associated with use/non-use (child age, respondent age, gender, and socio-economic status). In our CATI survey, 57% of parents said they “always” use a booster seat, and another 6.3% said they used one “frequently”. These self-reported rates of booster seat use exceed observed rates, confirming prior research (for example, [2,27]).

As is common for CATI surveys, there was some systematic bias in respondents. In particular, 77.6% had graduated from college or university compared to 53% of the Winnipeg population [28]. Only 5.5 percent of respondents earned less than the low-income cut-off, compared to about 10% of Winnipeg households [29]. These sampling biases may be related to data collection methods that rely on respondents that have a residential telephone number, and should be considered when interpreting results.

Given cultural standards of safety awareness and parental obligation to protect children, participants who attest to booster seat use may be reflecting social desirability [27,30]. As more regions pass booster seat laws, financial and legal fears may increase the importance of social desirability, and consequently weaken the utility of self-report instruments due to the bias against negative self-report. Indeed, social desirability bias has been found to be a key contributor to parents’ and caregivers’ positive report of use [31,32]. Consent bias may also contribute to inflated booster seat use estimates. Parents who do not use booster seats may be less likely to participate in the survey [12]. Finally, it is possible that some respondents do not understand the word always to mean 100% use [27]. For this reason, asking parents if they always use a booster seat could have captured instances where the rate of use was high, but less than perfect.

Telephone interviewing has advantages in that it is a good way to reach relatively large sample sizes, but in addition to the above issues, there may be considerable cost associated with this method, particularly if a project is focusing on a specific subset of the population. The challenge in the case of booster seat use (or any child-safety device), phone interviews must be conducted with parents/caregivers of children in the right age range. Our use of CATI surveys was negatively impacted by the high number of non-participants due to a lack of specificity in our telephone number directory. The vast majority of phone numbers called did not have a child in the specific age range. More focused dialing directories are available, but the high cost limits the feasibility of contacting a representative sample so that results can be more easily generalized to the population at large. Generalizability of phone interview results is also constrained by lower participation rates of low-income parents due to more limited access to cell phone and land-line phones.

In order to address these problems, we have also used face-to-face interviewing which has also been used successfully in other research on booster seat use [26]. We conducted in-person interviews using the same interview protocol originally designed for CATI with a much higher completion rate. The interviews were short, focused, and conducted at community events frequented by parents of children within the target range. When interviews were accompanied by a small incentive, such as a $5 gift card, participation rates were even higher. Parents were also more likely to permit interviews with their children in a face-to-face situation, as research assistants working in small teams provided formal identification and official informed consent forms which increased parent confidence. Other researchers have also had success with a short in-person surveys allow for detailed assessments of children’s ages, restraint use, and caregivers’ rationale for using or not using booster seats [13,26]. Our face-to-face interviews consistently showed that self-reported rates of booster seat use exceeded observed rates.

In evaluating the impact of child-safety campaigns and legislation, semi-structured focus groups can also be effective for a more in-depth understanding of both parent and child perceptions, rationales and beliefs concerning safely equipment (for examples, see [33,34]. Focus groups can be either homogenous or heterogeneous, designed to explore a wide variety of perceptions simultaneously or more in-depth perceptions of a particular group. For example, in our research we designed a focus group exclusively for new immigrants in order to increase their comfort level and to gauge if their perceptions concerning the use of safety equipment may differ from parents who were not new immigrants.

We have completed a number of focus groups with children and parents about their perceptions and use of booster seats, and these groups allowed the further exploration of topics such as whether parents actually think that booster seats are safer, whether children feel pressure from other children about using booster seats, and whether there are cultural differences in how booster seats are perceived. These are important areas to examine when designing and evaluating child safety campaigns and programs.

Conducting focus groups, however, is time- and labour-intensive and comes with its own set of challenges, including smaller sample sizes relative to telephone interviewing and observation. Challenges can be compounded when focus groups for children are involved, in terms of planning (parents/caregivers need to be available to provide consent), trust (especially when focus groups consist only of children without their parents in the room), and ethical considerations. These factors increase common challenges for conducting focus groups, such recruitment and scheduling.

We found that one of the key barriers for focus group participation was recruitment of participants in specific age subsamples. Challenges impacting participation include effectively advertising the groups and the importance of the study as well as transportation for potential participants. For this reason, focus groups were held in a variety of locations that were frequented by and therefore familiar to parents, such as community and recreation centres, museums, and libraries. Access to these sites increased the need for organization and relationship-building with local organizations. Such programs are likely to support the child-safety effort, and are fairly targeted with regard to the age range of children, which made it easier to ensure that the needed participants were found. With the partnership of a school, recruitment of lower-income families can be facilitated since school populations typically include families with a range of socio-economic backgrounds.

### 3.3. Bicycle Helmet Observations

We have observed the use of bicycle helmets annually since 1996. We have observed 190 Winnipeg sites each year, and in 2010 added 20 additional sites in order to further represent low-income areas. We have also observed helmet use at 54 rural sites (within 75 km of Winnipeg) and 68 sites in the rural-remote (more than 75 km from Winnipeg) areas. Observation sites included parks, schools, residential streets, major intersections, and cycling paths. Sites were chosen to represent geographic regions associated with published income indicators. Given the fact that bike riding tends to be closer to home (particularly for children), it is safer to assume that a child riding in a particular neighbourhood likely comes from a local household, and therefore from a particular socioeconomic group.

The fact that bicycle helmet observations can be conducted in neighbourhoods with particular income characteristics increases confidence that the majority of the children being observed are local, and provides important opportunities to examine socioeconomic effects on child safety. This is less true for booster seat observations, since people tend to drive beyond their local neighbourhood, and shop at central locations. It is also the case that, as income lowers, so does the probability of owning a vehicle or a bicycle in the first place. Our observations across Winnipeg have indeed shown a connection between bicycle helmet use and income. For example, in 2013, we observed that 31% of cyclists wore helmets in areas with an average household income below $42,556, while 72% of cyclists wore helmets in areas with an average household income above $65,945. We have found this difference to be incremental, with helmet use dropping steadily as average income declines.

Observation of bicycle helmet use may appear to be simpler than observation of booster seat use due to the higher visibility of children riding bicycles. While it may be clear whether a rider is wearing a helmet or not, it can be more difficult to assess whether the helmet is worn properly. Observers need to be trained regarding the proper tilt of the helmet as well as the proper location of the straps, which is also a key visible indicator of proper use. Recently, bicycle helmets have become available with static side straps, where the “V” shape over the ears does not have to be manually adjusted. The most common misuse of helmets is that they are often worn tilted too far backward, when the front of the helmet should only be two finger-widths above the eyebrows. Even with the need to identify proper use, however, roadside bicycle helmet observations faces fewer structural challenges than booster seat observations. This includes the identification of a child’s age, which can be based generally on the same developmental principles applied during booster seat observations, but with the advantage of seeing the entire body of the child.

Another advantage for bicycle helmet observations is the wider range of possible venues, such as parks, schools, residential streets, major intersections and cycling paths. As noted with booster seat observations, the potential bias of timing of observations is an important issue. Kraemer et al. (2014) found that helmet use was observed to be lower in the evening and on Fridays, which suggests that observation schedules must be carefully planned out to ensure that different times of day and days of the week are included equally.

A critical issue for bicycle helmet observation is weather. It has been illustrated that the number of cyclists observed is significantly correlated with predicted daily high temperature, chance of rain, and actual rain [35]. For this reason, a surveillance schedule should be flexible, particularly if resources are limited, since rainy days will be much less productive in terms of data collection.

For helmet surveillance, location is more sensitive than it is for booster seat surveillance because children do not tend to cycle as far from home as they are driven, and younger children in particular tend to stay close to home. Ridership is often recreational in nature, unlike booster seat usage. The social nature of bike riding is also a factor—children often tend to ride with others, including other children and their families. The more localized nature of bike riding can pose a challenge but also provides important opportunities. The challenge is that it is necessary to include a variety of locations that will capture recreational ridership as well as commuting (e.g., to school) in order to increase generalizability. For example, children’s recreational usage may be highest on popular pathways in parks, but their commuting usage may be highest near suburban schools. In our years of observation, helmet use has been most commonly observed on cycling paths and less often on residential streets. This is in sharp contrast to booster seat observations, which were most often observed in heavy vehicular traffic.

### 3.4. Bicycle Helmet Self-Report

As is the case for booster seat surveillance, focus groups and interviews have provided important information that complements observational data. Challenges for these focus groups are the same as those discussed above regarding booster seat focus groups. In the same way, such data collection allows for more detailed insight into the use of bicycle helmets as well. In our focus groups, for example, we found that most parents were aware of the helmet law in Manitoba but only about half were aware of the age requirement (helmets are mandatory until age 18). Recent immigrants were less likely to know about bike helmet laws and how to fit helmets properly. We also found that most parents are not aware of helmet fit guidelines. Focus groups with done with children indicated that most children use helmets frequently and prefer helmets that fit well. Children also talked about the design of helmets, including whether the helmet keeps the sun out of their eyes. The main reasons children gave for not wearing helmets were forgetting, peer pressure, and helmet fit. For teens, there was more concern about the helmet affecting their appearance (hairstyle) and their perception that helmets are not necessary when riding close to home. These are important insights that can inform the development and evaluation of safety campaigns.

Self-reported bicycle helmet usage information is also often collected as part of ongoing government surveys. For example, the Canadian Community Health Survey (CCHS) is an annual national cross-sectional survey collaboratively conducted by Statistics Canada, Health Canada, and the Canadian Institutes for Health Information. The CCHS collects data from Canadians aged 12 or older, with half of the interviews conducted in person and the other half by telephone. The survey includes one member of each household and cases are distributed proportionally across differing geographical regions in Canada. Because the CCHS uses a multi-stage sampling strategy and only 1% of the sample is identified using random-digit-dialing (RDD), the survey is not subject to the sampling biases that come with a strict reliance on RDD. Importantly, however, because the CCHS administers about half of the surveys over the phone and half in person, the telephone sampling biases discussed earlier regarding booster seat use data are reduced. In 2014, approximately four million Canadians aged 12 and older reported riding a bicycle within the past 12 months on the CCHS. Unfortunately, younger respondents under the age of 12 were not included.

## 4. Rural versus Urban Contexts

The distinction between urban and rural usage of safety devices is critical. Accurate surveillance in rural locations is especially important because unique intervention strategies may be needed to address the specific characteristics of rural living, such as longer distances from home to school and differing recreational activities [36]. Rural surveillance may be of greater concern in geographic regions with vast, sparsely-populated regions such as Canada’s Prairie Provinces or rural Australia, but these considerations can apply in any location. For example, the city of Winnipeg, with a population of approximately 800,000, contains about two thirds of the province of Manitoba’s population (1.3 million). Therefore, one third of the population would not be represented if rural surveillance were not conducted. Because of the low number of cases, rural results need to be considered with caution. Therefore, choice of location and consistency of observations over time becomes even more important.

Rural and urban bicycle helmet use rates have been found to be significantly different. Historically, helmet use rates are lower in rural areas [37], although the differences in use rates have been reversing, at least in Manitoba. Our observations have found that rural helmet use for children under age 18 is higher than helmet use in the city. For example, in 2015 we found that in Winnipeg, 59% of children and youth were wearing helmets, compared to 73% of those in rural areas and 71% of those in rural-remote locations. We have also found that bicycle helmet use by younger children (under age eight) is higher than it is for youth, and that in general, observed helmet use is about the same in rural areas as it is in the city.

Rural surveillance poses unique challenges, regardless of which methodology is used. For example, with regard to observation, the recruitment, training and supervision of field observers in rural areas is more difficult than in urban areas, particularly remote rural areas. Distance training for observers is one solution; we have trained observers remotely using the same photos used for local training accompanied by phone discussions. In addition, visitation by experienced observers to more remote sites to serve as supervisors for actual field training is also helpful. Because of the lower frequency of both vehicular and bicycle traffic, much more time is needed for observations at rural and remote sites in order to collect an adequate number of cases. There can also be systematic observation characteristics unique to rural and urban locations. For example, in our Winnipeg bicycle observation work, we have seen relatively few children, despite choosing locations near schools and residential streets, and regardless of the time of day or day of the week. In rural observations, the reverse is the case. There, the vast majority of our observations are young children with only a few youth and adults. This can generate problems when comparing urban and rural bicycle helmet rates, and could explain the fact that rural helmet use by children now seems to exceed urban use. Given these challenges and constraints, other forms of surveillance would make an important contribution to understanding bicycle helmet and booster seat use in rural and remote locations.

## 5. Emerging Technology

Technology provides several new surveillance methods for booster seat and bicycle helmet usage that can expand the ability to track safety device use and help address issues associated with rural regions. For example, Geographic Information Systems (GIS) have been used to examine injury rates and locate populations and regions at risk for particular injuries, as well as to assess access to traumatic care [38]. This method allows for special analysis of particular locations, and can be applied in layers. Specifically, it would be possible to map locations of traumatic presentations at hospitals and overlay that with bicycle helmet crashes and use rates across the same regions. This could help to determine the distance injured children must travel for treatment as well as for the relations between helmet usage rates and local treatment demand. This method could be of great importance for the analysis of rural areas, where health services are geographically spread out and people typically have to travel longer distances.

Smartphone applications are another increasingly popular method for collecting information. For example, QuickTapSurvey allows data collection in a survey format. Surveys can be sent out, but the software also uses the phone as a data-recording device where specific questions and response options can be programmed. Such a survey could be based on any data collection tool, whether responses to interview questions, focus group notes, or recording observations. All data can be directly stored into a database. This surveillance method would be more efficient than rural observational work, although it does depend on Internet access and participant compliance.

Recent research has compared the quality of survey interview data gathered using an automated smartphone system compared to traditional telephone interview. Researchers randomly assigned 634 survey respondents to four groups: one responding to an automated survey through texting, the second responding to an automated telephone interview, the third responding to a human telephone interview and the fourth responding to text messages from a human interviewer [39]. Data collected through texting was of a higher quality and, importantly, those responding by text were more likely to disclose sensitive information (such as sex partners and substance abuse) and showed a greater desire to participate in future interviews [39]. These researchers suggested that mobile devices may allow participants to respond where and when it is most convenient, and that automated systems may reduce social desirability concerns such as fear of being judged. Participation did not appear to be associated with factors such as gender, race and income and education, and completion rates were similar for all four groups.

Smartphone technology may be more appealing to children and adolescents than traditional phone or in-person interviews. In addition, parents may be more willing to disclose non-usage of safety devices for the reasons outlined above. Potential drawbacks may include longer time to completion for an interview, since a text interview has a different rhythm and can take much longer with long breaks between responses [39]. In addition, participants may be more likely to consult on their responses with others around them, making it impossible for researchers to know if confidentiality has been breached. Finally, texting may shorten detailed responses to open-ended questions that would be facilitated by an in-person interview.

Surveillance could be conducted more passively by simply collecting location and travel information from smartphones or other devices, such as fitness trackers, smart watches, etc. With consent, participants could volunteer to disclose their location and travel information, which can be tracked and stored on the phone. This information could then be compared to self-report or observation of booster seat or bicycle helmet use or non-use, or could incorporate photo sharing of how helmets are worn, and how booster seats are utilized. This approach would also be effective when studying rural regions. However, more research is needed on the validity and accuracy of these promising surveillance methods, and how they compare to more traditional observation and survey methods.

## 6. Conclusions

Given the importance and cost of injury prevention programs and campaigns such as community education, low cost distribution of helmets and booster seats, and social marketing strategies [40,41], accurate surveillance of the usage of safety devices is critical. Based on our field experience with booster seat and bicycle helmet surveillance over the past several years, we conclude the most rigorous surveillance approach is a multi-method combination of observation and self-report measures. It is important to ensure that each method is applied in a way that provides reliable and accurate results (by, for example, testing inter-rater reliability as discussed earlier). The objective of effective surveillance, however, is not to identify one ideal data collection method, but rather to use each method to provide part of the complete picture of the motivations, rationales, feelings and understandings that underlie the use or non-use of child safety devices. With a critical understanding of the challenges associated with each method, comprehensive surveillance campaigns can produce a complete and reliable overall picture. For the more objective numbers (for example, the number of children wearing helmets), it is important to ensure consistency in collection methods so that even if data is not perfect, year-over-year and region-to-region comparisons are valid.

In addition, there are critical considerations regarding the need to collect surveillance data in rural regions, especially since there seems to be systematic differences in safety device use in such settings. In order to perform surveillance methods in rural areas, it is important to develop and maintain ongoing relationships with schools, community centres and administrators in rural municipalities in order to build trust and support for child safety initiatives. Such relationships are critical to recruiting research assistants as well as focus group and interview participants. These relationships could include partnering to deliver public safety campaigns that, beyond improving surveillance, could lead to improved awareness and safety behavior in the communities. Clearly, future surveillance can be enhanced by incorporating promising new technology, as long as the validity and reliability of new measures is systematically assessed.

## Figures and Tables

**Table 1:** Comparison of booster seat use rates in Winnipeg with and without hybrid observations.

Year	2010	2011	2012	2013	2014	2015
Number	1720	1541	3235	1470	1941	1543
Hybrid Cases	176	294	490	285	618	454
Use Rate with all cases	15.3%	20.8%	31.6%	32.8%	33.7%	44.4%
Use Rate without hybrid cases	17.8%	25.7%	37.3%	40.6%	49.7%	63.0%
